# Kinetics of conductive hydro-drying of pumpkin (*Cucurbita moschata*) pulp

**DOI:** 10.1016/j.heliyon.2024.e36982

**Published:** 2024-08-28

**Authors:** Monica J. Ortiz-Jerez, Yendy X. Serna, y Jose E. Zapata

**Affiliations:** Nutrition and Food Technology Group, University of Antioquia, Colombia

**Keywords:** Conductive hydro-drying, Pumpkin, Cucurbita, Kinetics modelling, Effective diffusivity

## Abstract

Pumpkin (*Cucurbita moschata*) samples were dehydrated by conductive hydro-drying (CHD) (1 atm, 80 °C), sliced and purées, both structures with thicknesses of 1.5, 3 and 6 mm. Drying kinetics were analyzed and the effective diffusivity (*D*_ef_) was determined in both structures at the three thicknesses. Drying curves were fitted to ten kinetic models: Lewis, Henderson & Pabis, Logarithmic, Page, Wang & Singh, Page Modified, Midilli, Diffusion Approximation, Two-term Exponential and Verma. *D*_ef_ was determined by analytical solution of Fick's Second Law in rectangular coordinates by Crank's method. In general, the semi-empirical model that best fit showed was Midilli's model. However, the importance of phenomenological models such as the analytical solution of Fick's second law for process scaling and equipment design should be considered. These modeling results aid in predicting performance and fine-tuning hydrodrying processes for sustainable, high-quality food. Future applications may involve integrating these models into industrial-scale hydrodryers, reducing energy consumption and environmental impact.

## 1 Introduction

Pumpkin (*Cucurbita moschata*) has been grown for over 6000 years all over Latin America, with presence in low-altitude areas, hot weather and high humidity [1]. It is a vegetable with a high potential for industrial applications due to its nutritional contents and facility to grow, but the few applications assessed to give it added value limit its offer to commercialize fresh product in different kinds of markets, and serve different cuisine or other purposes [2]. Pumpkin in Colombia is a crop with little demand compared to other vegetables, as it is not widely popular or accepted because of the few applications forms it has. However, its importance in food safety at national level, and the growing demand of new raw materials for agroindustry, have encouraged research on this vegetable [1]. From a nutritional perspective, pumpkin pulp has a high water content (80–96 g/100 g), and low carbohydrate concentrations (4.6–5.6 g/100 g), protein (0.6–1.8 g/100 g), fat (0.0–0.20 g/100 g), and fiber (0.5–1.3 g/100 g), in addition to a significant value in antioxidants, vitamin A (280 mg/100 g), and vitamin C (12 mg/100 g) [3]. Its pulp has been reported to have a phenolic compounds content of 236 ± 1.8–1113 ± 4 mg GAE/100 g b s., and 72.9 ± 2.2 mg GAE/100 g b s., respectively, both fresh and dehydrated. Likewise, with provitamin A in the form of total carotenoids of 490.1–1365.8 μg/g, and 141.5 ± 1.32 mg/100 g of sample, respectively, both fresh and dehydrated [4].

Refractance Window drying uses hot water at about 90 °C as the energy source and an infrared transparent plastic (Mylar^TM^) as the contact surface for drying [5]. Early hypotheses about the heat transfer mechanism in this process suggested that wet material in a thin plastic film over hot water creates a "window" for infrared radiation and, as the material dries, the "window" gradually cuts off the radiation. A conjugate model of mass and heat transfer demonstrated that a large portion of thermal energy is transferred by conduction [6,7]. These authors proposed the term “conductive hydrodrying (CHD)” for a technology in which the radiation heat transfer component is reduced by obstructing the refraction window using a gray body. Subsequently, energy transmission occurs mainly by conduction, in a way that can be used to dehydrate samples with thicker samples [6].

One of the alternatives to give added value to pumpkin is dehydration, which improves its conservation by reducing water activity, thus preventing the growth of microorganisms and reducing the speed of chemical alterations. In this way, the pumpkin has been dehydrated using hot air [3,4], a method that has been applied in some cases to obtain flour [2]. Solar drying [8], fluidized bed [9], microwaves and microwaves combined with hot air [10] have also been used. However, the negative effects of heat on nutritional and sensory characteristics have led to the evaluation of innovative and improved drying methods for this vegetable, such as osmotic dehydration combined with hot air [11], refractance window drying (RW^TM^) - [12] and freeze-drying [2], among others. Nevertheless, some of these methods present some problems to be implemented at an industrial level, such as solar drying; others may be too expensive, such as freeze-drying and vacuum drying; require subsequent drying, such as osmotic dehydration, which produces intermediate moisture products [11]; or have process limitations, such as the RW technique, which is efficiently applied to thin films (below 1 cm) [6].

The industrial application of drying as an operation that ensures the quality of the dehydrated product and that, in addition, makes it viable from an economic point of view, requires establishing the mechanisms that govern the behavior of the system during the process. Diffusional migration of moisture contained in a product is important in the drying of several materials, including foods [13]. This non-steady state mass transfer is traditionally studied using the solution of Fick's second law, considering the food as a moist and porous solid, to obtain moisture diffusion coefficients [14]. To analyze the kinetics of drying fruits and vegetables, there are mathematical models that describe the simultaneous phenomena of mass and heat transfer in a non-steady state [3,15]. These models not only help to establish the final moisture content of the products, but also to estimate the ideal time and temperature required for the process; they also make it possible to predict storage and packaging conditions, design new drying systems or improve existing ones.

The diffusion mechanism of a species in the liquid phase is extremely complex due to the diversity in the chemical composition and physical structure of food matrices, such as fruits and vegetables. The determination of effective diffusivity (D_ef_) also includes the material-water interactions of the vegetable matrix, the volume change associated with the porosity and tortuosity of the sample and multiphase water transport [5]. The integrated form of Fick's second law is normally used to determine D_ef_. The analytical solutions are applied considering several assumptions, including that the size and geometry of the material remain constant and, at a given temperature, the diffusion coefficient is independent of the moisture content. Although these two assumptions are not met in practice, analytical solutions with average diffusivity satisfactorily reproduce the moisture evolution. Crank (1975) provides detailed solutions to the diffusion equation when the diffusivity is considered constant or concentration-dependent for several initial and surrounding conditions [13].

There are several thin-layer models to describe the food drying process, which can be grouped into theoretical, semi-theoretical (Lewis, Page, modified Page, Henderson & Pabis, Logarithmic, Two-term, Two-term exponential, Diffusion approximation, Verma, Midilli, etc.) and empirical ones (such as Wang and Singhś) [16]. Some of them have successfully adapted to the drying of several cucurbita species such as Page, Logarithmic, Verma and Midilli [3,17]. The kinetics of drying pumpkin with hot air, whether in an oven, trays or tunnel, has been studied mainly to determine the mass transfer, the energy necessary for drying, and the effect on the sample thickness [17]. However, the kinetic modeling of conductive hydro-drying has not been studied so far in pumpkin (Cucurbita moschata). Therefore, the purpose of this study is to determine the most appropriate thin-layer model to understand the behavior of conductive hydrodrying (CHD) of pumpkin (slices and purées) in different thicknesses and to establish the effective diffusion coefficient resulting from each product presentation.

## 2 Materials and methods

### 2.1 Preparation of raw material

Pumpkin (*Cucurbita moschata*) were used in medium ripening stage grown in the rural area of La Pintada (Caldas, Colombia), and purchased in a local market in Medellín (Antioquia, Colombia). Fresh pumpkins weighed approximately 3.5 kg, with a soluble solids content of 5.27±1.7 °Brix and an initial moisture content of 92.23±1.85 % (wb). It was washed with alkaline detergent and then disinfected with citrosan (0.2 %), peeled, and cut in halves. Initial moisture and dry solids content were determined from the fresh pulp using the method of progressive heating infrared scale (Shimadzu, ATX224 Model, Japan), and the concentration of total soluble solids (SS), by using a refractometer (Milton Roy Company® Model LR 45227, USA), expressing data as Brix degrees (°). All measurements were taken by triplicate. Once the pumpkin is open, the pulp was separated from the placental cavity (seeds and strands) to adequate it in two forms: slices and purées. Slices were obtained with a sharp stainless-steel blade mandoline slicer (Simposh, SP15C Model, USA), sliding the flat section of the fruit to obtain 1.5, 3, and 6 mm thick rectangular pieces. The length of the pieces was adjusted manually using a knife set. To obtain pumpkin purée, a food processor was used (Magic Bullet, NB-101S Model, USA), processing for 1 min until obtaining a homogeneous paste.

### 2.2 Conductive hydro-drying (CHD)

**Fig. 1:**
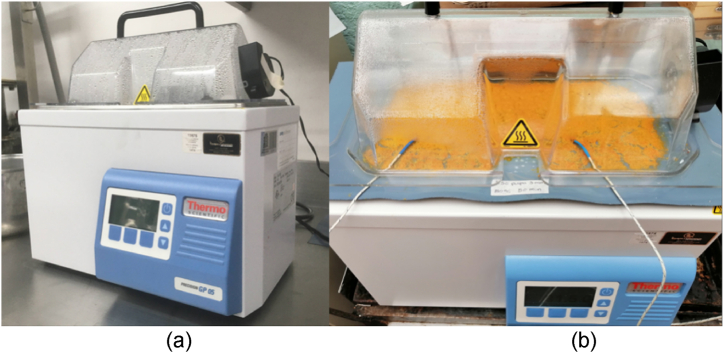
Current view of conductive hydro-drying equipment (a) water bath and (b) pumpkin samples on the plastic film.

Experimental runs were carried out using a stainless-steel thermostatic water bath (Thermo Scientific TSGP05 Model 0.1 °C precision, USA) with 5 L of capacity and a 15.4 × 30 cm^2^ (long x wide) transfer area (Fig. 1). The bath, filled with tap water, was covered with a 0.250 mm thick polyester film (Mylar™) (D type, DuPont, USA), previously covered with multi-surface matt black paint (Pintuco, Colombia) on one of its sides, making sure the side painted of film touches the water as seen in Fig. 1. Mylar is a transparent plastic used tipically in the RW technology because of its optical and heat radiation transfer properties. By obstructing the passing infrared radiation from the water, energy transmission will mainly ocurrs by conduction [6]. The average thickness of the painted film was determined at various film regions with a Vernier caliper (Ubermann, Sodimac, Colombia), resulting in an average value of 0.3 ± 0.02 mm.

The bath temperature was controlled at 80+2 °C. For each test, approximately 25 g of pumpkin sample (in slices and purées) of 1.5, 3, and 6 mm thick were disposed, spread evenly on the plastic film. The thickness of slices and processed pulp (purée) was measured in four points with the Vernier caliper. Once the samples were placed, before each run, the thermostatic bath was covered using a customized lid. This system has an extractor installed on the inside of the equipment lid that continuously removes the evaporated water from the samples, thus preventing condensation. During the CHD, weight loss of the samples was registered on an analytical scale (Shimadzu, TXB2201L Model, Japan) every 10 min until obtaining a constant moisture content and below 15 % (wb). After drying, the product was tempered for 10 min at lab conditions (RH: 68 % and T: 24 °C), packed in re-sealable plastic bags, and kept in a desiccator. All experiments were carried out in triplicate.

### 2.3 Drying curves

The Moisture Ratio (MR), and the drying rate of the pumpkin samples during drying were calculated using equation (1) [3].

(1) $$\text{MR}=\frac{X_{t}‐X_{\text{eq}}}{X_{0}‐X_{\text{eq}}}$$

where, X_t,_ is the moisture content at any time t; X_0_, the initial moisture content; and X_eq_ the moisture content in the equilibrium all expressed on a dry base (g water/g dry solid). Drying curves were drawn from experimental data expressed in MR vs t. Dry solids mass was determined from the initial moisture content of the sample. The moisture content in the equilibrium corresponds to the drying for a very long period when the equilibrium with the surrounding atmosphere was achieved [3]. It was considered to be 0.01 g water/g dry solid.

### 2.4 Estimation of water diffusion coefficient

Considering pumpkin as a wet solid in fine, infinite slices, where water loss occurs only perpendicular to the heat transfer surface, and assuming that initial moisture distribution in the samples is uniform, and shrinking is negligible, the effective diffusion coefficient was determined using the differential equation based on Fick's second law (equation (2)) for water diffusion in non-stationary state [13]:

(2) $$\frac{\partial X}{\partial t}=D_{\text{ef}}\frac{\partial^{2}X}{{\partial z}^{2}}$$

where X is the total moisture content of the sample per unit of dry solid (g water/g dry solid); D_ef_ is the effective moisture diffusivity (m^2^/s); t is drying time (s); z is the coordinate in the direction of mass transfer (m).

For rectangular coordinates and negligible external resistance, equation (2) can be analytically solved by applying Crank's solution [18]:

(3) $$\text{MR}=\frac{X_{t}‐X_{\text{eq}}}{X_{0}‐X_{\text{eq}}}=\frac{8}{\pi^{2}}\sum_{n=0}^{\infty }\frac{1}{{\left(2n+1\right)}^{2}}\exp\left(\frac{{\left(2n+1\right)}^{2}\pi^{2}}{4}\text{Fo}\right)$$

where Fourier's number (*Fo*) is given by *Fo* = D_ef_ t/L^2^, being L the product thickness (m). For long enough times (*Fo* > 0.2), the first term of the infinite series provides a good estimate of the solution to equation. (3) [3,19]. Despite the drying took some minutes for some products, (mainly those of 1 mm thick), the definition of the *Fo* (dimensionless) value showed accuracy by truncating the infinite series in the first term. Taking only the first term of the infinite series, equation (3) results in:

(4) $$\text{MR}=\frac{X_{t}‐X_{\text{eq}}}{X_{0}‐X_{\text{eq}}}=\frac{8}{\pi^{2}}\;\exp\left(‐2.47\text{Fo}\right)$$

*Fo* value was estimated using the non-linear optimization code GRG (Generalized Reduced Gradient) of Excel (Microsoft, 2019 version) for each treatment. By plotting *Fo* versus drying time (t), a linear adjustment was obtained. Finally, the effective diffusion coefficient (D_ef_) of each product was estimated from the slope of the straight line.

### 2.5 Modeling of drying kinetics

Ten kinetic models of thin layer were selected to adjust to the drying curves to find the most suitable model that describes the CHD of pumpkin (*Cucurbita moschata*).

The models selected searched to include theoretcal models as Lewis, Henderson and Pabis, Logarithmic, Page, modified Page, Midilli, Diffusion Approximation, Exponential, Two-term, and Verma; and empirical model as Wang & Singh. The authors and bibliographic sources of these models were widely referenced in Gomes et al. (2018) [16] and Mphahlele et al. (2019) [20].

A non-linear regression analysis was carried out using the minimum square method, with Excel (Microsoft, 2019 version). The goodness of fit of each model to describe the kinetics of CHD of pumpkin was determined using statistical parameters such as correlation coefficient (R^2^), the reduced squared chi (*χ*^2^), and the root-mean-square error (RMSE) [16,21].

## 3 Results and discussion

### 3.1 Moisture content and drying time

**Fig. 2:**
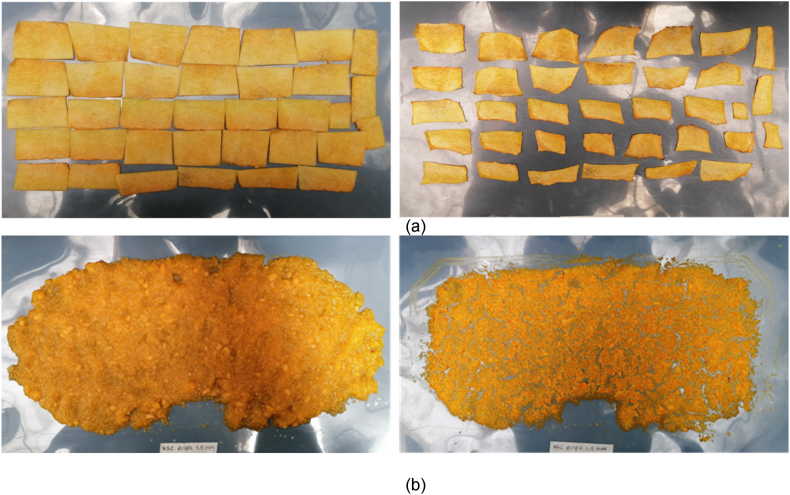
Current view of the pumpkin samples before (left) and after (right) conductive hydro-drying CHD (a) slices, and (b) purée.

The samples arranged on the drying sheet are shown in Fig. 2 before and after drying according to the type of presentation (slices and purée).

**Fig. 3:**
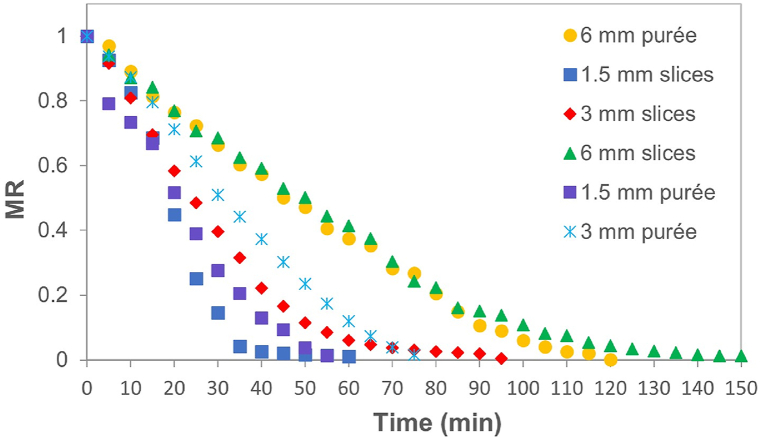
Variation of the dimensionless moisture ratio (MR) with conductive hydro-drying (CHD) time of pumpkin at 80 °C in slices, and purée.

Fig. 3 shows the experimental changes of the adimensional Moisture Ratio (MR) concerning the drying time at the CHD temperature (80 °C) for slices and purées in the three thicknesses studied. The curves are comparable with other pumpkin studies with a typical behavior of the drying curves at different temperatures (between 30 and 110 °C), drying methods (convective, solar, vacuum, microwave), structures (pulp, mashed, grated) [17], and geometrical shapes such as slices [22], cubes [9,15], cylinders [3,23]. The reduction in the Moisture Ratio (MR) is proportional to the weight loss of the sample. Therefore, the low values of MR indicate slight changes in the registered weight of the samples in time. Until achieve values near to moisture of equilibrium. At this point, it is assumed that the free water is eliminated almost completely. Therefore, moisture content in the equilibrium could be considered as the lowest possible obtained in each treatment [14].

When comparing drying curves for the thicknesses of 1.5, and 3 mm, it can be noticed that at the same test temperature (80 ± 2 °C), there is a lower difference that between the 3 and 6 mm samples. The increase in the Moisture Ratio (MR) in the thinner slices is due to the air movement on the surface of the pumpkin layer, which is faster in a smaller surface area than in a larger. It can be inferred that, at the same temperature, thickness does not have much effect on drying velocity at lower thicknesses [21]. This was also observed in purées. Due to the fluid structure of the pulp, it is expected that moisture evaporation occurs at the same speed with which water reaches the surface. Therefore, the effect of thickness lacks has no importance on the dimensionless moisture ratio (MR) [14].

The initial free moisture (X_t_ -X_eq_) of the samples was 11.12 ± 0.04 (db). At the end of the CHD, it was observed that, for each thickness of 1.5, 3, and 6 mm, the slices had final moistures of 0.040 ± 0.01, 0.167 ± 0.001, and 0.013 ± 0.01 (db), respectively, with drying times of 60, 95, and 150 min, respectively. This indicates that, with each 100 % increase in the thickness of the sample, there is an increase of 58 ± 2 % in drying time. On the other hand, final moistures of the purée in each thickness were 0.121 ± 0.10, 0.125 ± 0.09, and 0.221 ± 0.003 (db), with drying times of 60, 75, and 120 min, respectively. With this data, it can be observed that, when CHD ends, the purées keep a higher moisture than the slices. By increasing purée thickness in 100 % (from 1.5 mm to 3 mm), the sample showed a 25 % increase in drying time, whereas for the 6 mm, it increased in a 60 % regarding the 3 mm, like what happened with the slices. By increasing thickness of samples, a proportional increase in drying times is expected during the period of constant drying velocity, which occurs while the solid surface is very wet at first and there is a continuous water film onto it [14]. By continuing the drying, during the decreasing drying time, time continues to increase due to the difficulty to eliminate moisture.

Data show the facility or difficulty to extract available water (free water) for the CHD of each structure and treatment. Therefore, 6 mm slices and pulp showed evident differences. That can be attributed to the fact that the solid in pulp is more porous, and weaves and clefts on its surface help to obtain a higher velocity than it would have a completely flat surface. This means that most water that evaporates comes from inside [14]. The shape of drying curves of slices is different to those of purées (Fig. 3), as the latter show minimum change in slope, it is indicate that all the drying is produced in the constant drying period [14]. This is possible as long as water keeps reaching the surface as fast as it evaporates [14], thanks to the absence of membranes that may keep the water trapped. On the other hand, studies of slices of different materials, including pumpkin, have shown that there is no constant drying period, and the whole process takes place during the decreasing drying period [21,22].

### 3.2 Kinetic modeling and non-linear adjustment

The drying process is based on simultaneous phenomena of heat and mass transfer, in which water is transferred mainly by diffusion from inside the food to the surface [15]. The liquid and/or steam diffusion mechanism is extremely complex due to the diversity in chemical composition and the physical structure of the different products [3].

Table 1 shows the values of the parameters and statistical coefficients of the models studied for 1.5 mm thick samples. Correlation coefficients are in bold, in which the four best range adjustments between 0.994 and 0.999 were observed. In the case of slices, there is a better adjustment with Midilli, Page, Verma, and Wang & Singh models, whereas for purée, the best models were Wang & Singh, Midilli, Verma, and Diffusion Approximation.

**Table 1:** Modelling of drying in thin layer for pumpkin of 1.5 mm slices and purée at 80 °C.

Model	Parameter	slices 1.5 mm				purée 1.5 mm			
		Value	R^2^	*χ*^2^	RMSE	Value	R^2^	*χ*^2^	RMSE
Lewis MR = exp(-k^.^t)	k	0.049	0.974	0.010	0.101	0.042	0.984	0.005	0.069
Henderson & Pabis MR = a^.^exp(-k^.^t)	k	0.054	0.971	0.008	0.089	0.044	0.982	0.004	0.066
	a	1.152				1.055			
Logarithmic MR = a^.^exp(-k^.^t) + c	k	0.045	0.974	0.006	0.080	0.023	0.995	0.001	0.034
	a	1.214				1.380			
	c	−0.084				−0.380			
Page MR = exp(-k^.^t^n^)	k	0.001	**0.999**	0.000	0.016	0.010	0.993	0.002	0.040
	n	2.231				1.428			
Wang & Singh MR = 1+a^.^t + b^.^t^2^	a	−0.034	**0.983**	0.005	0.070	−0.030	**0.996**	0.001	0.029
	b	0.000				0.000			
Modified Page MR = exp(-(k^.^t)^n^)	k	0.220	0.974	0.010	0.101	0.194	0.984	0.005	0.069
	n	0.220				0.216			
Midilli MR = a^.^exp(-k^.^t^n^) + b^.^t	k	0.001	**0.999**	0.000	0.014	0.010	**0.996**	0.001	0.030
	a	0.974				0.957			
	b	0.000				−0.001			
	n	2.391				1.368			
Diffusion Approximation MR = a^.^exp(-k^.^t)+(1-a)^.^exp(-k^.^b^.^t)	k	0.049	0.974	0.010	0.101	0.010	**0.995**	0.001	0.033
	a	0.343				−1.845			
	b	1.000				1.824			
Two-term Exponential MR = a^.^exp(-k^.^t)+(1-a)^.^exp(-k^.^a^.^t)	k	0.049	0.974	0.010	0.101	475.510	0.984	0.005	0.069
	a	1.000				0.000			
Verma MR = a^.^exp(-k^.^t)+(1-a)^.^exp(-g^.^t)	k	0.111	**0.994**	0.002	0.039	0.015	**0.995**	0.001	0.033
	a	18.928				10.949			
	g	0.120				0.013			

**Fig. 4:**
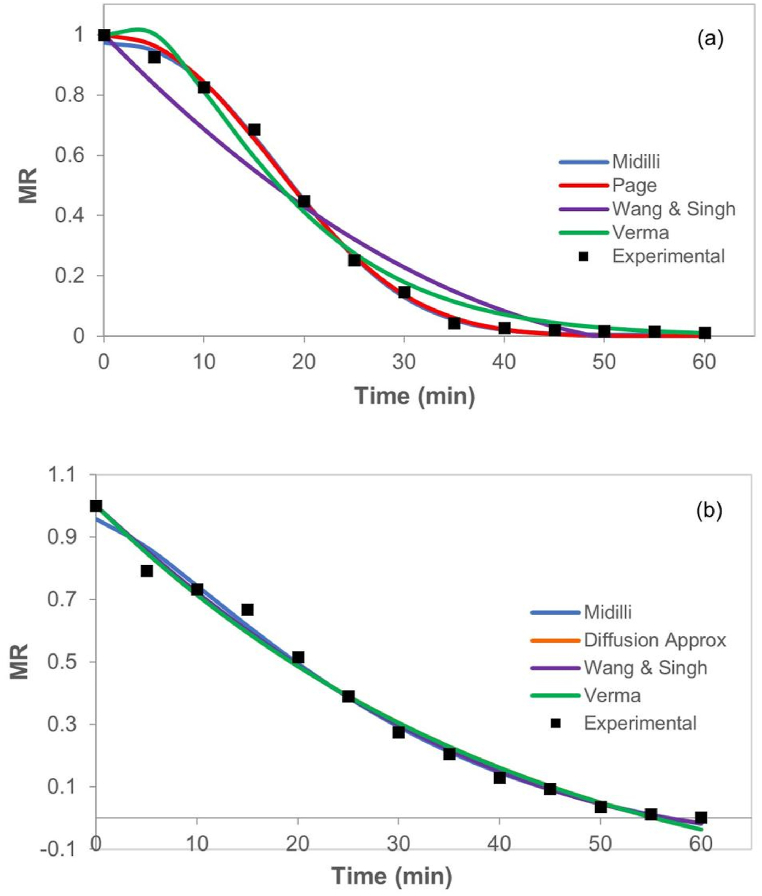
Dimensionless Moisture Ratio (MR) versus time (t) of conductive hydro-drying CHD of pumpkin in 1.5 mm thick (a) slices, and (b) purée.

The drying kinetics of 1.5 mm thick samples in slices and purées, respectively, are shown in Fig. 4 (a, and b), contrasted with the curves simulated by the four models that better adjusted for this thickness in each type of products. The shape of the curves is like those observed in other studies on pumpkin drying for different structures and low-thickness geometries [21,22], the study of different drying methods, and pumpkin rehydration (*C*. *Moschata Duch*) [23].

**Fig. 5:**
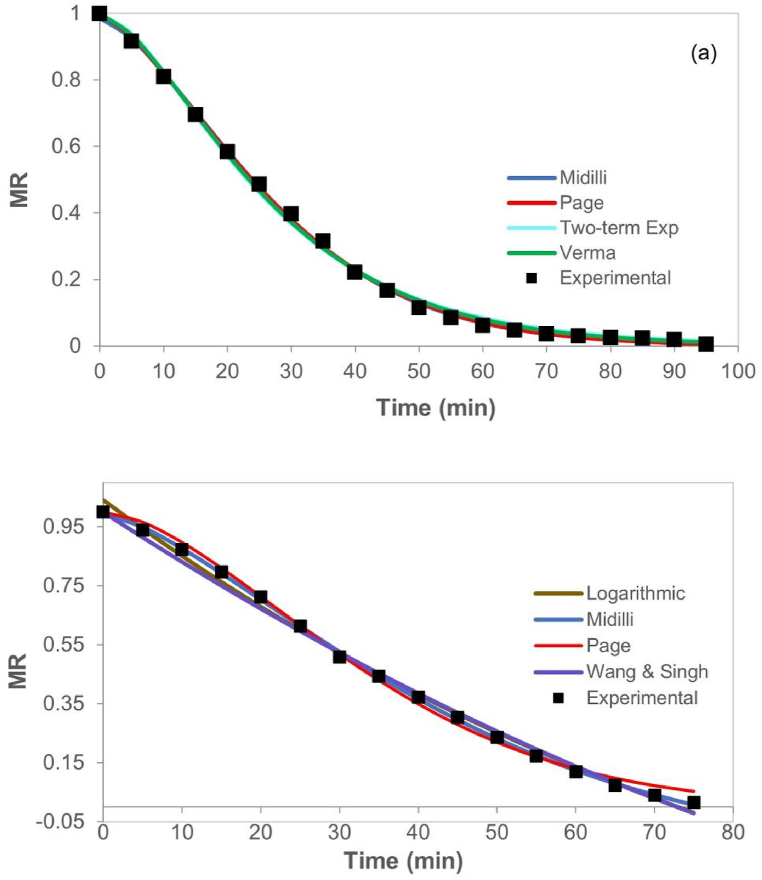
Dimensionless Moisture Ratio (MR) versus time (t) of conductive hydro-drying (CHD) of pumpkin in 3 mm thick (a) slices, and (b) purée.

Table 2 has the parameters of each model and the goodness of fitness of all CHD treatments for pumpkin in both 3 mm structures. Likewise, Fig. 5 (a, and b) show pumpkin drying kinetics of slices and purées (3 mm), respectively, contrasted with the curves simulated by the four models that best fitted for this thickness in each type of products. The coefficient of the best four models was in the range between 0.998 and 1.000. For slices (Fig. 5 a), the results show that the models with the best adjustment were Midilli, Page, Verma, and Two-term exponential. Whereas for purée (Fig. 5 b), the best models were Midilli, Page, Logarithmic, and Wang & Singh. This indicates that Midilli's model is the best option to adjust treatments applied to 3 mm samples.

**Table 2:** Modelling of drying in thin layer for pumpkin of 3 mm slices and purée at 80 °C.

Model	Parameter	slices 3 mm				purée 3 mm			
		Value	R^2^	*χ*^2^	RMSE	Value	R^2^	*χ*^2^	RMSE
Lewis MR = exp(-k^.^t)	k	0.034	0.993	0.003	0.058	0.026	0.984	0.004	0.060
Henderson & Pabis MR = a^.^exp(-k^.^t)	k	0.038	0.991	0.002	0.048	0.029	0.979	0.003	0.051
	a	1.101				1.118			
Logarithmic MR = a^.^exp(-k^.^t) + c	k	0.030	0.994	0.001	0.035	0.010	**0.998**	0.000	0.013
	a	1.168				2.056			
	c	−0.099				−1.016			
Page MR = exp(-k^.^t^n^)	k	0.007	**1.000**	0.000	0.010	0.003	**0.998**	0.000	0.017
	n	1.451				1.640			
	n	1.451				1.640			
Model	Parameter	Value	R^2^	*χ*^2^	RMSE	Value	R^2^	*χ*^2^	RMSE
Wang & Singh MR = 1+a^.^t + b^.^t^2^	a	−0.025	0.998	0.001	0.023	−0.017	**0.998**	0.000	0.014
	b	0.000				0.000			
Modified Page MR = exp(-(k^.^t)^n^)	k	0.053	0.993	0.003	0.058	0.153	0.984	0.004	0.060
	n	0.652				0.170			
Midilli MR = a^.^exp(-k^.^t^n^) + b^.^t	k	0.006	**1.000**	0.000	0.009	0.004	**1.000**	0.000	0.004
	a	0.986				0.994			
	b	0.000				−0.001			
	n	1.498				1.477			
Diffusion Approximation MR = a^.^exp(-k^.^t)+(1-a)^.^exp(-k^.^b^.^t)	k	0.034	0.993	0.003	0.058	0.032	0.985	0.002	0.045
	a	0.342				1.218			
	b	1.000				86.783			
Two-term Exponential MR = a^.^exp(-k^.^t)+(1-a)^.^exp(-k^.^a^.^t)	k	0.052	**0.999**	0.000	0.017	494.907	0.984	0.004	0.060
	a	1.978				0.000			
Verma MR = a^.^exp(-k^.^t)+(1-a)^.^exp(-g^.^t)	k	0.067	**0.999**	0.000	0.014	0.004	0.997	0.001	0.026
	a	20.190				10.014			
	g	0.070				0.003

The drying curves shown in Fig. 5 keep the same behavior observed in 1.5 mm samples (Fig. 4). In general, differences in shape and their slopes are evinced according to the type of structure, indicating that the evaporative resistance is higher in slices than in purée. Therefore, slice drying does not take place in a constant drying period, possibly due to the presence of membranes that keep water trapped during the whole process, avoiding drying velocity to be maximum [21]. To the slices, the slop can change from 0.02 to 0.0014 min^−1^, while to purée the slope remain almost constant about 0.013 min^−1^.

**Fig. 6:**
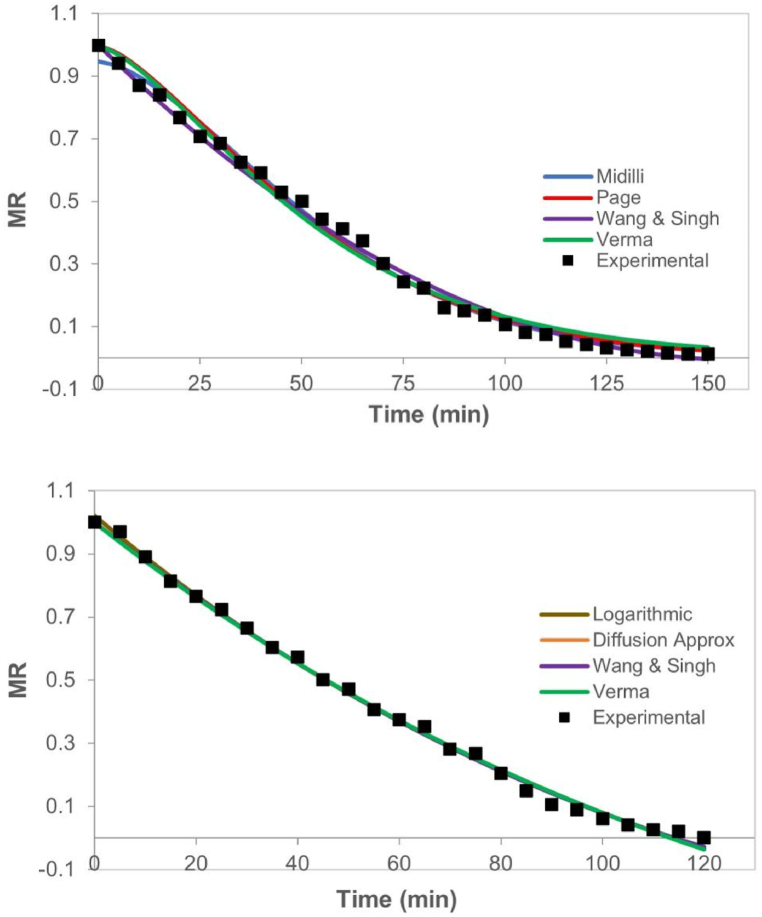
Dimensionless Moisture Ratio (MR) versus time (t) of conductive hydro-drying (CHD) of pumpkin in 6 mm thick (a) slices, and (b) purée.

The parameters of kinetic and adjustment models of pumpkin in 6 mm slices and purée are summarized in Table 3. Fig. 6 (a and b) correspond to the CHD kinetics of samples for both structures, contrasted with the curves simulated by the four models that fit best for this thickness in each type of product. The coefficient of the best four models was in the range between 0.996 and 0.999 For slices, the models that showed best fitting were Wang & Singh, Midilli, Page, and Verma; and for purée, the best models were Wang & Singh, Logarithmic, Verma and Diffusion Approximation. This shows that Wang & Singh's model provides the best fit for the experimental data of 6 mm samples CHD. However, for purée samples, Midilli's model ranks within the five best fittings with a correlation factor (R^2^) higher than 0.995. The drying curves shown in Fig. 6 have the same behavior seen in 1.5- and 3-mm samples (Fig. 4, Fig. 5, respectively), keeping the differences between both structures.

**Table 3:** Modeling of drying in thin layer for pumpkin of 6 mm slices and purée at 80 °C.

Model	Parameter	slices 6 mm				purée 6 mm			
		Value	R^2^	*χ*^2^	RMSE	Value	R^2^	*χ*^2^	RMSE
Lewis MR = exp(-k^.^t)	k	0.018	0.990	0.004	0.065	0.018	0.987	0.005	0.073
Henderson & Pabis MR = a^.^exp(-k^.^t)	k	0.019	0.986	0.003	0.057	0.020	0.982	0.004	0.063
	a	1.098				1.103			
Logarithmic MR = a^.^exp(-k^.^t) + c	k	0.012	0.995	0.001	0.031	0.008	**0.998**	0.000	0.018
	a	1.278				1.705			
	c	−0.240				−0.683			
Page MR = exp(-k^.^t^n^)	k	0.003	**0.997**	0.001	0.026	0.003	0.995	0.001	0.033
	n	1.447				1.481			
Wang & Singh MR = 1+a^.^t + b^.^t^2^	a	−0.013	**0.998**	0.001	0.022	−0.012	**0.999**	0.000	0.017
	b	0.000				0.000			
Modified Page MR = exp(-(k^.^t)^n^)	k	0.038	0.990	0.004	0.065	0.128	0.987	0.005	0.073
	n	0.466				0.142			
Midilli MR = a^.^exp(-k^.^t^n^) + b^.^t	k	0.001	**0.998**	0.001	0.023	0.002	0.996	0.001	0.031
	a	0.947				0.964			
	b	0.000				0.000			
	n	1.601				1.558			
Diffusion Approximation MR = a^.^exp(-k^.^t)+(1-a)^.^exp(-k^.^b^.^t)	k	0.006	0.996	0.001	0.031	0.003	**0.998**	0.000	0.019
	a	−11.594				−4.069			
	b	1.097				1.663			
Two-term Exponential MR = a^.^exp(-k^.^t)+(1-a)^.^exp(-k^.^a^.^t)	k	1290.636	0.990	0.004	0.065	885.741	0.987	0.005	0.073
	a	0.000				0.000			
Verma MR = a^.^exp(-k^.^t)+(1-a)^.^exp(-g^.^t)	k	0.033	**0.996**	0.001	0.030	0.004	**0.998**	0.000	0.019
	a	15.149				9.866			
	g	0.035				0.003

Several papers have shown pumpkin drying using other drying methods and similar thin-layer models. However, very few studies have gone deeper in low-thickness samples (<2 mm), which is important due thin layer drying assume that at each instant there is no potential gradient in the direction of transfer. For example, Yaldýz and Ertekýn (2001) [8] used twelve models to study the behavior of solar drying for pumpkin and other vegetables in slices, and found that the diffusion approximation model showed the highest R^2^ value for pumpkin. In this current study, this model showed to be one of the best four fittings for 1.5 mm purée samples. On their side, Amer (2011) [24] showed results of 12 kinetic models for RW drying at 80 °C of very low-thickness mango purée (0.3 mm) and, based on several statistical parameters, selected the two-term, Diffusion approximation, Verma, and Logarithmic models as the best ones to represent the thin-layer behavior of the product. Likewise, Azizi et al. (2016) [25] defined that the best fitting for RW drying at 80, 90, and 100 °C of very thin slices of starfruit (0.8, 1.6, and 2.4 mm), is the Logarithmic model.

Hashim et al. (2014) [21] studied the behavior of convective drying of low-thickness (2–4 mm) pumpkin (*C. moschata*) slices at temperatures between 50 and 70 °C, with three models: Lewis, Henderson & Pabis, and Page. The models that best fitted providing determination coefficients higher than 90 % were Lewis, and Henderson & Pabis, whereas Page's model was the worst (R^2^ < 70 %). On the other hand, Guiné et al. (2011) [3] out of the six models tested to theoretically adjust the experimental data of convective drying of 4 mm circular slices of pumpkin (*C. maxima*) between 30 and 70 °C, evinced that Page and modified Page's models are the best to reproduce the experimental results, whereas Wang & Singh's model was the worst. Yacanto et al. (2018) [17] compared the drying on stove and IR thermobalance of pumpkin (*C. moschata Duch.*) anco type in cylinders, mashed, and grated (3 mm) at 65 °C, and found that, in the case of cylinders, Page's model was the best; for mashed, it was Parabolic (MR = a +bt + ct^2^); and for grated, it was Midilli's. On this current study, Page's model showed high correlation coefficients, mainly for CHD of slices (1.5, 3, and 6 mm), with values of 0.999, 0.999, and 0.997, respectively. In the case of purées, Page's model was the second best for the 3 mm thickness. Henderson & Pabis model did not qualify among the four best, but did show correlation coefficients in the range of 0.971–0.991 for all samples and treatments. In contrast, Midilli's model had the first place for 3 mm slices and purées, and the second for both 1.5 mm presentations, showing its feasibility to adjust the CHD of samples considered as low and middle-thickness (2–5 mm).

The analysis of fruit and vegetable drying kinetics in high-thickness slices or pulps (≥5 mm) has been mainly focused on the determination of the effective diffusion coefficient D_ef_ [4,13,23]. However, application of the thin-layered modeling in thick samples has been scarcely used in pumpkin and other food matrixes. Alibas (2007) [10] studied hot air, microwave, and combined drying at 50 and 75 °C of 5 mm thick pumpkin slices (*Cucurbita maxima*), adjusting all treatments to the Page model with outstanding results, giving correlation coefficients of 0.998–0.999. In another paper, Sojak and Głowacki (2010) [9] using a variety of giant pumpkin (*Justynka* – 957), cut in 10 mm slices and cubes, determined the drying kinetics at 80 °C with natural and forced convection in tunnel and fluidized bed driers during two stages or periods. Authors propose a model that represents each of the periods taking into account shrinking, which is used for finding a proper fitting despite they do not include observations of the shape changes of the dried pieces.

For products other than pumpkins, Dissa et al. (2011) [19] fitted direct solar drying curves using 10 mathematical models for slices of two varieties of mango (Amelie and Brooks) cut in 8 mm slices, being the Two-term, Verma, and Diffusion approximation the ones with the highest R^2^ values (0.994–0.999). The semi-empirical models of Newton (Lewis), Henderson & Pabis, Page, and Two-terms were used for adjusting the experimental data in the convective drying modeling of unripe Cardaba banana slices (3, and 7 mm) between 30 and 60 °C [26], it was found that, out the first three, Page's is the one that reproduces best, but only the two-term model describes data well enough at all experimentation temperatures, both in thin and thick samples. Unlike Hii and Ogugo (2014) [27], who showed that starfruit drying of 5 mm slices in a convective oven (60°- 80 °C), that previous underwent bleaching and sugar solution, Page's model could describe the moisture diffusion process during drying. Whereas Mphahlele et al. (2019) [20], showed that the behavior of convective drying in thin layer (at 40, 50, and 60 °C and constant velocity) of 5 mm or larger samples of pomegranate fruit peel, Midilli's model showed the best-fitting among 10 models tested, with a 0.999 coefficient. Unlike the above, Olawoye et al. (2017) [28] showed Wang & Singh's model is the best among 12 models selected for the hot air drying analysis (50, 60, and 70 °C) of unripe banana in 5 mm slices. This current study, in thick 6 mm slices, as well as for other authors, Page and Midilli's are among the four best models, whereas for 6 mm purées, these two models do not classify. Table 3 shows how Wang & Singh's model better represents experimental data for both structures.

There is no previous reference of studies on kinetic modeling of pumpkin CHD. However, in a test of refractance window (RW) drying at 70 °C of papaya (*Carica papaya* L) purée at 2, 3, and 4 mm, between Newton (Lewis) and Midilli's Models, it was found that the latter is the most suitable to predict experimental curves [29]. This was also observed by Ortiz and Ochoa-Martínez (2014) [6] during RW drying of pumpkin parallelepipeds (10 mm thick, 90 °C) with a determination coefficient of 0.999. In another RW drying test assisted with an infrared lamp (60–90 °C) for low-thickness purées (3 mm) of golden berries, Puente-Díaz et al. (2020) [30] determined that the best thin-layer model is Midilli's, by comparing experimental data with the predictions among five models, which could be a useful tool to describe pumpkin drying kinetics, and the most suitable to estimate drying time under different RW drying conditions [30]. In contrast with these results, Azizi et al. (2016) [25], selected the Logarithmic model as the one with the best fit for RW drying at 80, 90, and 100 °C of starfruit slices (0.8, 1.6, and 2.4 mm), among five models studied (Newton, Logarithmic, Page, modified Henderson & Pabis, and Two-term exponential). Nevertheless, Midilli's model was not included in their study.

Such diversity of results is mainly due to the different drying types, the conditions used in each drying, and their effects on the response variables studied according to each case. However, it is easy to notice that some semi-empirical methods used here and also observed by other researchers, tend to refer better acceptance and response in terms of fitting and correlation. According to the results obtained in this study, and based on the acceptance of other studies, it can be said that the two best models that represent CHD for 1.5 mm pumpkin slices are Page and Midilli's, and for 1.5 mm purée are Wang & Singh's and Midilli's. For 3 mm slices and purée, the best models are Midilli and Page's. For 6 mm slices, Wang & Singh and Midilli's models stand out, and for 6 mm purée, Wang & Singh and Logarithmic. Although the 10 models studied favorably adjust to the experimental curves with superior correlation coefficients, Midilli (with 4 terms), Page (with 2 terms), and Verma's (with 3 terms) models showed enough adjustment to the experimental data. The *k* drying constant is common for the three models. It ranges between 0.010 and 6x10^−4^ for Midilli; 0.010 to 0.001 for Page; and 0.011 to 0.004 for Verma. In most cases, it tends to decrease with the increase of the thickness of the sample at the same temperature both for slices and pulp. This was also observed by Zalabaga and Carballo (2015) [26] in slices, and by Gomes et al. (2018) [16] in purées of other materials rather than pumpkin. However, as these authors state, its magnitude is affected by the drying temperature. The other parameters (a, b, n, and g) are not common to the three models mentioned and therefore, they are not compared here. It has been reported that the introduction of *n* parameter in Page's model has a significant importance in the adjustment [26]. In short, statistical analysis parameters showed that Midilli's model is the one that best fits for most CHD treatments. That may be due to the presence of four terms, which provide a better approximation to drying curves with exponential tendency [20]. However, Wang and Singh's model can be recommended for thicker samples considering the results obtained for 6 mm slices and purée.

### 3.3 Water diffusion coefficient

The resulting effective diffusion coefficients (D_ef_) of the products studied according to each treatment at 80 °C, appears in Table 4, which were within the range of 2.78x10^−10^ and 4.78x10^−9^ m^2^/s.

**Table 4:** Effective diffusion coefficients (D_ef_) of pumpkin as a function of structures and thicknesses.

Type	1.5 mm		3 mm		6 mm	
	D_ef_ (m^2^/s)	R^2^	D_ef_ (m^2^/s)	R^2^	D_ef_ (m^2^/s)	R^2^
Slices	6.53x10^−10^	0.922	1.35x10^−9^	0.947	4.98x10^−9^	0.924
Purées	2.78x10^−10^	0.923	3.15x10^−10^	0.923	6.00x10^−10^	0.929

Several researchers have reported similar orders of magnitude results for pumpkin drying by different methods, mainly convective drying of slices in a temperature range between 30 and 70 °C [4,22]. However, in similar temperature ranges, other researchers reported higher D_ef_ values (in magnitude order of 10^−8^ and 10^−7^ m^2^/s) for sliced pumpkin [3], and in cubes and cylinders [23]. Effective diffusivity has also been reported in a wide array of products in slices or purées, all in magnitude orders between 10^−10^ and 10^−7^ m^2^/s, and at hot air drying temperatures between 40 and 90 °C, such as potato and carrot cylinders [13]; starfruit slices [27]; green banana slices [26,28], and agroindustrial residues such as pomegranate fruit peel [20]. Additionally, the D_ef_ has been reported for some low-thickness purées dried using RW drying, such as mango [5,24], Carica papaya [29], and golden berries [30], all of them in a magnitude order of 10^−10^ to 10^−9^ m^2^/s. The values obtained in this study for pumpkin slices and purées are within the general range of 10^−11^ to 10^−8^ m^2^/s, reported for most food materials [22,27].

It is also observed that the D_ef_ of products of both structures increase when sample thickness is increased. This behavior has been previously reported [5,16,19,26], and in all cases, it is associated to the effect of the drying temperature increase. Nevertheless, in this current study, all experiments were carried out at the same water bath temperature (80 ± 2 °C), and the tendency is preserved. When a homogeneous material mass undergoes drying, the water mass flow generated favors water displacement toward the material surface, thus being vaporized and, as a consequence, effective diffusion increases [4]. Diffusivity represents the velocity with which water displaces from the inside to the material surface to evaporate [16]. This velocity seems to be affected by the shrinking kinetics. As the samples dries, it shrinks and, consequently, it is more difficult for water to diffuse in thinner samples [5]. Additionally, this phenomenon could be related to the facts that the D_ef_ for samples in purées are lower than for slices. Initial availability of water in the surface of purées could favor drying in less time compared with slices (Fig. 5, Fig. 6), thus developing the reduction in its dimensions while drying. When shrinking, moisture transfer rate may be reduced, which reflects on lower diffusivity.

At the experimental temperature, thinner samples dry faster due to their higher specific surface area. Thicker samples require longer drying times or may require higher temperatures. This is because heat must penetrate deeper into the sample to evaporate water and therefore the moisture removal process takes longer. Distributing samples evenly on the drying surface is essential to allow for even heat distribution and faster drying. The pureed product allows it to be spread in a thin and uniform layer on the drying sheet while the slices tend to separate from each other and shrink. It is important to consider sample thickness when designing hydro-drying processes to ensure optimal quality and efficiency, depending on texture, storage, reconstitution, or rehydration requirements.

It is to notice that the model based on the effective diffusivity determination (D_ef_) has lower adjustments that empirical or semi-empirical models. However, it must be considered that this is a phenomenological model based on transference models that rule the drying process, which can explain if better. Additionally, it is useful to extrapolate results and escalate the process, while empirical and semi-empirical processes have limitations in this aspect.

### 3.4 Limitations and new features

The drying rate in the CHD is influenced by the thickness of the product. Increasing the thickness of the product or reducing the temperature of the hot water can dramatically decrease the drying rate. This can be a limitation when processing products with different thicknesses. Although CHD better retains heat-sensitive nutrients, the specific drying kinetics for CHD have not been widely investigated. This could be a limitation in terms of process optimization.

The novelty of this work focuses on the study of the conductive hydro-drying kinetics of the pumpkin, a highly thermosensitive plant source, rich in carotenes and vitamins, and there is not a similar study about it. The study was based in phenomenological analysis of moisture loss using Fick's Second Law and ten semi-empirical to determine which of them are a good fit. These modeling results aid in predicting performance and fine-tuning hydrodrying processes for sustainable, high-quality food. Future applications may involve integrating these models into industrial-scale hydrodryers, reducing energy consumption and environmental impact.

To contribute to its implementation in food processing and preservation, future studies in CHD are proposed as follows: Optimize the different process parameters (such as water temperature or drying time) in the efficiency of CHD. Optimal conditions can improve drying rate while maintaining product quality. Another possible study is to explore alternative heat sources beyond hot water. For example, use microwave-assisted CHD or combine it with other energy-efficient technologies. Evaluate quality and shelf life studies of dry products. Investigate changes in nutritional content, flavor, color and texture. Carry out comparative studies with other conventional drying methods (such as hot air drying or freeze drying). Evaluate energy consumption, nutrient retention and overall product quality. Assess the economic feasibility of implementing CHD on an industrial scale. Factors such as equipment costs, energy efficiency and environmental impact can be considered. Finally expansion the applications to other specific foods (for example, other vegetables, fruits, herbs or meat) and tailor CHD processes to their unique characteristics.

## 4 Conclusions

Pumpkin (*Cucurbita Moschata*) kinetics behavior during conductive hydro-drying can be successfully modeled with any of the models used in this study. The best fits are given by semi-empirical models, where Midilli model stands out. The phenomenological model based on Fick's second law, despite of not have the best adjustments, must be considered, because the effective diffusion coefficient is a parameter used in the design of equipment and scale-up of process. The best treatment corresponds to CHD of the slices at 1.5 mm because they take less time in reach the equilibrium moisture. However, in general sense, the purées have better kinetic behavior than sliced samples at any observed thicknesses.

## Ethics statement

The current study doesn't include human or animal assays.

## Data availability statement

Publicly available datasets were analyzed in this study.

Data will be made available on request.

## CRediT authorship contribution statement

**Monica J. Ortiz-Jerez:** Writing – review & editing, Writing – original draft, Project administration, Formal analysis, Data curation, Conceptualization. **Yendy X. Serna:** Methodology. **y Jose E. Zapata:** Writing – review & editing, Writing – original draft, Conceptualization.

## Declaration of competing interest

The authors declare that they have no known competing financial interests or personal relationships that could have appeared to influence the work reported in this paper.

## Figures and Tables

**Table:** 

*Symbols*	
*X*_t_	Moisture content at any time t, (g water/g dry solid)
*X*_0_	Initial moisture content (g water/g dry solid)
*X*_eq_	Moisture content in balance (g water/g dry solid)
*X*	Total moisture content of the sample per dry solid unit (g water/g dry solid)
*D*_ef_	Effective diffusion coefficient of moisture (m^2^/s)
*t*	Drying time (s)
*z*	Perpendicular distance to mass transfer (m)
*Fo*	Fourier number given by *F*o = *D*_ef_*t*/*L*^2^
*L*	Product thickness (m)
*Abbreviations*	
CHD	Conductive Hydro-drying
RW	Refractance window
GAE	Galic Acid Equivalent
wb	wet base
db	dry base
